# Première expérience de l'utilisation du Misoprostol comme soin après avortement (SAA) à Libreville, Gabon

**DOI:** 10.11604/pamj.2014.18.301.4309

**Published:** 2014-08-14

**Authors:** Sosthène Mayi-Tsonga, Ulysse Minkobame, Arielle Mbila, Pamphile Assoumou, Ayisha Diop, Beverly Winikoff

**Affiliations:** 1 Université des Sciences de la Santé de Libreville, Libreville, Gabon; 2 Projet FIGO pour la lutte contre les avortements à risque au Gabon; 3 Service de Gynécologie Obstétrique du Centre Hospitalier Universitaire de Libreville, Libreville, Gabon; 4 Gynuity Health Projects, New York City, New York, USA

**Keywords:** Avortement, avortement incomplet, traitement médical de l'avortement, soin après avortement, misoprostol, Gabon, abortion, incomplete abortion, medical treatment of abortion, post abortion care, misoprostol, Gabon

## Abstract

**Introduction:**

Une étude a été menée afin de déterminer le taux d'acceptabilité de 400µg de misoprostol par voie sublinguale comme traitement de première intention de l'avortement incomplet et de préciser le taux d'avortement complet ou vacuité utérine.

**Méthodes:**

Les femmes éligibles avaient un diagnostic clinique d'avortement incomplet avec une taille utérine inférieure à celle d'un utérus de 12 semaines d'aménorrhées (SA). Chacune a reçu 400µg de misoprostol par voie sublinguale. Les femmes ont été revues après une semaine. A J7, celles qui n'avaient pas complètement expulsé ont eu le choix entre une nouvelle consultation de suivi à J14 et la pratique d'une évacuation chirurgicale immédiate.

**Résultats:**

145 patientes ont été éligibles et ont toutes accepté la méthode (100%). L’âge moyen était de 25,9 ± 6 ans. A J7, 120 patientes étaient guéries (85,7%). A J14, le taux de réussitea été de 95,7% soit 134 patientes guéries. Les patientes guéries ont déclaré être très satisfaites (57,5%), satisfaites (41,8%) et insatisfaite (0,7%). Au total,128 femmes (95,5%) ont dit être prêtes à utiliser de nouveau le misoprostol comme méthode d’évacuation utérine en cas d'avortement incomplet.

**Conclusion:**

L’étude démontre que le 400µg misoprostol par voie sublinguale nous permet de prendre en charge d'une manière adéquate l'avortement incomplet surtout dans les pays à faible ressource et notamment dans les structures sanitaires de première ligne ou éloignées.

## Introduction

Un progrès a été accompli en termes de réduction de la mortalitématernelleau Gabon d'après le rapport de l'EDSG-II de 2012 [1]. Le taux est passé de 519/10^5^naissances vivantes (NV) en 2000 à 316/10^5^NV en 2012 [1]. D'après ce rapport [1], le taux de mortalité maternelle liée aux avortements à risque esten baisse. En 2009, nous faisions déjà ce constat à partir des chiffres hospitaliers [2]. Nous avons également constaté que les femmes gabonaises utilisent, maintenant, plus souvent le misoprostol pour avorter [2, 3]. Cependant, les délais d'initiation des soins après avortement (SAA) demeuraient trop longs dans notre service jusqu'en 2010 [4]. Pour pallier à ces retards, nous avons introduit l'aspiration manuelle intra utérine (AMIU) depuis 2012. Les résultats ont été appréciables dès la première année avec une réduction significative des délais de SAA, passés de 23,1 heures en moyenne à 1,2 heure [5]. Dans le souci de réduire d'avantage ces délais, nous avons décidé d'introduire le misoprostol comme traitement médical de première intention des avortements incomplets dans notre service. L'objectif principal de la mise en place de cette nouvelle stratégie thérapeutique était de contribuer d'avantage à la réduction de la mortalité maternelle au Gabon. Les objectifs spécifiques étaient de déterminer le taux d'acceptabilité de ce SAA, de décrire le profil épidémiologique des femmes qui l'ont accepté, de préciser le taux d'avortement complet et de déterminer le délai moyen de prise en charge des patientes.

## Méthodes

**Type et cadre de l’étude:** Il s'agit d'une étude prospective, descriptive et analytique, conduite dans le service de Gynécologie Obstétrique du Centre Hospitalier Universitaire de Libreville (CHUL) sur 6 mois, du 15 avril 2013 au 15 octobre 2013. Libreville est la capitale du Gabondont la population est de 1,5 million d'habitants. Près de 60% de ces habitants vivent à Libreville. La maternité du CHUL, centre de référence national, est la plus grande de tout le paysavec une moyenne de 8500 accouchements annuels environ 8% d'avortements pris en charge annuellement.

**Critères d'inclusion:** Ont été incluses, les femmes admises à la maternité du CHUL pour une complication d'avortement et qui remplissaient les critères suivants: avoir des saignements vaginaux, un col ouvert lors du toucher vaginal, une taille utérine correspondant à un terme ≤12 semaines d'aménorrhée (SA), sans signes de choc hémorragique ou infectieux et ayant donné un consentement écrit. Pour les femmes qui ne remplissaient pas ce critère, le SAA a été soit l'aspiration manuelle intra utérine (AMIU), soit le curetage.

Les patientes qui remplissaient les critères d'inclusion, ont bénéficié d'une information éclairée sur le protocole et reçu gratuitement une dose de 400µg unique de misoprostol (Misoclear^®^, ACME; Inde) par voie sublinguale, soit deux comprimés de misoprostol200µg placés sous la langue pendant environ 30 minutes. L'heure d'arrivée et d'initiation du traitement ont été consignées sur le dossier de suivi. La patiente a été gardée en observation pendant deux heures afin d'identifier et de notifier la survenue d’éventuels effets secondaires immédiats. Puis, elle a été revue à J7 afin d'apprécier l’état de vacuitéde l'utérus. Cette confirmation reposait sur des critères essentiellement cliniques (arrêt des saignements, fermeture du canal cervical, arrêt des douleurs pelviennes), parfois sur l’échographie pelvienne de contrôle qui mesurait la baisse du volume des débris utérins au mieux, la vacuité utérine. Dans le cadre du suivi à domicile, un appel téléphone a été effectué à J2 pour éliminer les lignes de gravité dûment mentionnés sur la fiche de consentement remise à la patiente. En cas de nécessité, la femme devait se rendre immédiatement à l'hôpital. Lorsque l’évacuation utérine n’était pas complète à J7, il était donné le choix à la femme d'attendre une semaine supplémentaire pour voir si l'avortement se complétait ou une évacuation chirurgicale immédiate (AMIU) lui était proposée. Si la patiente acceptait le premier choix, elle était revue une semaine après (J14 après la prise du misoprostol). L'AMIU était systématique lorsque les saignements persistaient 14 jours après l'administration dumisoprostol. La supplémentation en fer et la prescription d'une antibiothérapie se faisait au cas par cas.

### Collecte et analyse des données

Elle a été réalisée à partir des dossiers d'enquête individuelle. Les données suivantes ont été recueillies pour chaque patiente: caractéristiques socio démographiquesdélai de prise en charge, avortement complet à J7 et J14. La variable dépendante était l'avortement complet à J7. Les variables indépendantes ont été le niveau scolaire (instruites/non), la parité (0/ ≥ 1), la gestité (0/ ≥ 1) et le terme gestationnel (<8/ ≥ 9 SA). Les données ont été saisies sur le logiciel Excel de Windows7 puis analysées sur le logiciel SPSS 18.0. Les variables quantitatives ont été exprimées en moyennes avec leur écart-type et les modalités qualitatives ont été rendues en pourcentage. Celles-ci ont été comparées grâce aux tests de Khi-2, du Khi-2 de Yates et du Test Exact de Fisher. Le p-value a été fixé à moins de 5%. Le protocole a reçu un avis favorable du Comité National d'Ethique et de la Recherche du Gabon et le protocole est enregistré sous la référence interne PROT No008/2013/SG/CNE.

## Résultats

Cent quarante-cinq patientes ont été éligibles au protocole. Toutes les 145 patientes ont accepté le misoprostol (100%) L’âge moyen était de 26 ans avec des extrêmes de 14 et 47 ans, la majorité des femmes était célibataires (83,4%) et avait un niveau d’éducation au-delà du secondaire (86,4%), confère Tableau 1. Les patientes étaient en majorité paucipares (n ≤ 3 pares) soit 70%. Le terme gestationnel moyen était de 9,1 ± 2,2 SA avec des extrêmes de 5 et 12 SA. Cinq femmes ont été perdues de vues, et ne sont pas inclues dans l'analyse finale du taux de réussite. Le délai moyen de prise en charge thérapeutique entre l'arrivée des patienteset l'administration du traitement était de 35 ± 10,6 minutes (médiane 32,2 min) avec des extrêmes de 20 et 85 minutes.

**Tableau 1:** Caractéristiques des participantes

	n = 145
Age: médiane, moyenne ± ET (variance)	26 ± 6,1 (14-47)
**Niveau de scolarisation:% (n)**	
Aucun	4,1 (6)
Primaire	7,6 (11)
Secondaire	72,4 (105)
Universitaire	15,9 (23)
**Situation familiale:% (n)**	
Célibataire	83,4 (121)
Marié	16,6 (24)
Parité: médiane, moyenne ± ET(variance)	1,6, 1,9 ± 1,7 (0-10)

Il y a eu 5 cas de perdues de vue (3,4%) et 140 autres (96,6%) ont terminé l’étude. Parmi elles, 134 (95,7%) l'ont terminée exclusivement avec du misoprostol alors que 6 (4,2%) ont eu recours à l'AMIU (Tableau 2). Parmi les 140 patientes ayant terminé l’étude, l’évaluation clinique exclusive a suffi pour dire que l'avortement était complet chez 138 patientes (98,6%). Elle n'a pas été suffisante dans 2 cas (1,4%) où le recours à l’échographie a été nécessaire. A J7, 120 patientes avaient totalement arrêté de saigner et ont donc été déclarées guéries. Le taux d'avortement complet à J7 a été de 85,7% (Tableau 2). A J14, 14 patientes (70%) parmi les 20 patientes non guéries à la première visite présentaient, elles aussi, une vacuité utérine complète. Le saignement persistait seulement chez 6 patientes (30%) et elles ont eu une AMIU. Au total, 134 patientes (95,7%) étaient considérées guéries à J14 (Tableau 2).

**Tableau 2:** Résultats

	N = 140^a^(%)
Avortement complet J7	85,7 (120)
Avortement complet J14	95,7 (134)
Intervention chirurgicale	4,2 (6)
Délai de prise en charge médiane, moyenne ± ET	32,2, 35,4±10,6

a 5 femmes perdues de vue

En définitive, le taux de réussite globale de la méthodea été de 95,7% soit 134 patientes guéries,de J7 à la seconde visite de suivi. La proportion des paucipares qui étaient guéries à J7 n'a pas été statistiquement différente de celle des multipares guéries également à J7 (p = 0,15). Ce constat a été le même entre les paucigestes et les multigestes (p = 0,48) ainsi qu'entre les patientes dont le terme gestationnel était ≤9SA et celles de terme ≥10 SA (p = 0,44). Pour les 134 patientes guéris exclusivement par le misoprostol, la durée moyenne d'avortement complet ou vacuité utérine était de 5,1 jours + /-2, 3 avec des extrêmes de 1 et 13 jours (médiane 4,8 jours). Le pic de fréquence était entre 4 et 7 jours (82,8%). Parmi les 134 patientes guéries par le misoprostol, 77 (57,5%) ont déclaré être très satisfaites, 56 (41,8%) étaient satisfaites et 1 (0,7%) n’était pas satisfaite du traitement. Les effets secondaires ont été rares et la majorité des femmes les ont trouvés tolérables ou facilement tolérables (86,6%) comme le montre le Tableau 3. Au total, 128 femmes (95,5%) ont dit être prêtes à utiliser de nouveau le misoprostol comme méthode d’évacuation utérine en cas d'avortement incomplet. De même, 130 femmes (97%) ont déclaré qu'elles recommanderaient cette méthode à une amie pour l’évacuation utérine après un avortement incomplet (Tableau 3).

**Tableau 3:** Effets secondaires et acceptabilité du traitement ; (n)

Effets secondaires	n = 134^a^
Douleurs abdominales	73,1 (98)
Fièvre/frissons	32,1 (43)
Diarrhée	28,4 (38)
Vomissements/nausées	24,6 (33)
Céphalées	23,1 (31)
Vertiges	0,7 (1)
Sévérité des effets secondaires	
Très sévère / sévère	8,2 (11)
Tolérable / facilement tolérable	86,6 (116)
Pas tolérable	5,2(7)
**Acceptabilité de traitement**	
Niveau de satisfaction	
Très satisfaites / Satisfaites	99,3 (133)
Non satisfaites	0,7 (1)
**Difficulté du traitement**	
Pas du tout difficile	70,9 (95)
Un peu difficile	23,1 (31)
Difficile	6,0 (8)
Choisir méthode dans le future en cas de besoin	95,5 (128)
Recommanderait à une amie	97,0 (130)

a Entretien de sortie non disponible pour 6 femmes

## Discussion

En seulement 6 mois de mise en place, et malgré les fortes réticences de certains autres personnels au début de notre étude, cette méthode semble être bien acceptée par les patientes. Malgré la limitation d'un échantillon restreint, ce taux élevé d'acceptabilité est identique à ceux de Dabash [6] et Diop [7] qui ont retrouvé des taux de 96,8% et de 98%, en comparant respectivement le misoprostol à l'AMIU^6^ et la voie orale à la voie sublinguale [7]. L'intérêt lié à l'utilisation du misoprostol pour le traitement des avortements incomplets a été étudié dans des contextes et pays variés tels qu'aux États-Unis [8, 9], au Royaumes-Unis [10] et dans des pays disposant de ressources limitées comme le Burkina Faso [11, 12], le Ghana [13], la Tanzanie[14] et le Nigéria[15]. Ces auteurs [8–15] ont démontré que le misoprostol pouvait constituer une alternative au traitement chirurgical de l'avortement incomplet, avec des taux de réussite allant de 94,5% à 99%. Comparativement à l'AMIU, le misoprostol est une méthode sure et efficace. Dao [11]a retrouvé un taux d'avortement complet de 94,5% avec 600 µg de misoprostol versus 99,1% avec l'AMIU. Schwekerela[14] a retrouvéun taux de 99% pour le même dosage de misoprostol versus 100% pour l'AMIU. De même, pour certains auteurs [9, 11, 16], la dose de 400µg de misoprostol par voie sublinguale est aussi efficace que 600 µg par voie orale et avec moins d'effets secondaires. Il n'y a pas eu de relation d'association entre la parité, la gestité, le terme gestationnel et l'efficacité à J7. Ces caractéristiques n'influencent pas la qualité de la contractilité du myomètre. C'est essentiellement la pharmacocinétique du misoprostol, la voie d'administration et la dose qui conditionnent le taux d'efficacité [17]. Plusieurs études montrent des taux d'avortement complet dès le 7^ème^ jour, identiques au notre (87%). Diop [7] a retrouvé 80% d'avortement complet à J7 parmi les 300 cas inclus. Pour Dao [11], ce taux d'avortement complet à J7 était de 94,5% parmi les 221 femmes prises en charge. Dans l’étude de Taylor [13], 98% des 230 femmes traitées ont été guéries dès la première visite.

Notre souci permanant étant la réduction des délais des soins obstétricaux et néonataux d'urgence (SONU), le délai moyen d'initiation du misoprostol que nous avons retrouvé nous conforte pour plusieurs raisons. D'une part, parce que ce délai a été réduit de moitié par rapport à celui retrouvé par l’étude de Mayi [5] en 2012 qui avait relevé un délai moyen de 1,4 heure pour l'AMIU. Ce délai de 1,4 heure était déjà très appréciable comparativement au délai moyen, 23,1 heures, retrouvé en 2009 par ce même auteur[4] dans le même service. D'autre part, parce que dans l'espoir de nous rapprocher des objectifs du millénaire pour le développement de 2015 (OMD-5), ce délai moyen de 35 minutes est fort encouragent.

Le misoprostol comme traitement médical des avortements incomplets peut être une stratégie opérationnelle bénéfique pour notre service, en particulier, et pour notre pays, en général. Les cas étudiés par Mayi [4] avaient été tous traités par curetage à la curette au bloc opératoire. Or, la surcharge d'activité dans les salles opératoires ne permettait pas d'optimiser la qualité et la disponibilité des SAA [18], allongeant ainsi les délais de prise en charge hospitalière. Ces retards sont dûs aux dysfonctionnements de nos services obstétricaux africains et c'est justement ce que nous souhaitons améliorer dans notre service. Lee [19] a étudié l'impact psychologique et la satisfaction des patientes suite au traitement médical des avortements spontanés. Celui-ci a permis de conclure que le traitement médical par le misoprostol est psychologiquement acceptable et compatible aux croyances ethno médicales des patientes. Notre taux de satisfaction est superposable à ceux retrouvés par Shochet [20] et Thieba [12]. Le misoprostol est acceptable par les femmes comme moyen d’évacuation utérine.

## Conclusion

Cette étude a retrouvé une efficacité de 95,5%, un délai moyen de prise en charge de 35 minutes et une grande satisfaction des patientes. Le misoprostol est une méthode sûre et acceptable pour traiter les avortements incomplets du premier trimestre. La simplicité de l’équipement nécessaire et de la procédure technique en font une méthode particulièrement adaptée aux pays à faible ressource et notamment aux structures sanitaires de première ligne ou éloignées.

## References

[1] Direction Générale de la Statistique (DGS) (2012). Enquête Démographique et de Santé de 2012. http://www.stat-gabon.ga.

[2] Mayi-Tsonga S, Diallo T, Litochenko O, Methogo M, Ndombi I (2009). Prévalence des avortements clandestins au centre hospitalier de Libreville, Gabon. Bull Soc PatholExot..

[3] Mayi-Tsonga S, Diallo T, Litovchenko O, Metogho M, Ndombi I (2009). Etude comparée des complications des avortements clandestins: Misoprostol versus autres méthodes abortives. Clin Mother Child Health..

[4] Mayi-Tsonga S, Litochenko O, Ndombi I, Diallo T, De Sousa M-H, Faundes A (2009). Delay in the provision of adequate care to women who died from abortion-related complications in the principal maternity hospital of Gabon. Reprod Health Matters..

[5] Mayi-Tsonga S, Assoumou P, SimaOlé B, Bang J, Meyé JF, Faundes A (2012). The contribution of research results to improvement abortion care in Libreville. Reprod Health Matters..

[6] Dabash R, Ramadan MC, Darwish E, Hassanein N, Blum J, Winikoff B (2010). A randomized controlled trial of 400(g sublingual misoprostol versus manual vacuum aspiration for the treatment of incomplete abortion in two Egyptian hospitals. Int J GynecolObstet..

[7] Diop A, Raghavan S, Rakotovao JP, Comendant R, Blumenthal PD, Winikoff B (2009). Two routes of administration for misoprostol in the treatment of incomplete abortion: a randomized clinical trial. Contraception..

[8] Venture Stratégies Innovations (2012). Vies maternelles sauvées grâce au misoprostol: Soutien politique et institutionnel. VSI Revue.

[9] Blum J, Winikoff B, Gemzell-Danielsson K, Ho PC, Schiavon R, Weeks A (2007). Treatment of incomplete abortion and miscarriage with misoprostol. Int J Gynecol Obstet..

[10] American College of Obstetricians and Gynecologists - ACOG Committee Opinion N° 427 (2009). Misoprostol for Post abortion Care. Obstet Gynecol.

[11] Dao B, Blum J, Thieba B (2007). Is misoprostol a safe, effective and acceptable alternative to manual vacuum aspiration for postabortion care? Results from a randomized trial in Burkina Faso, West Africa. BJOG..

[12] Thieba B, Adama Z, Ouattara (2012). Oral misoprostol as first-line incomplete abortion in Burkina Faso. Int J GynecolObstet..

[13] Taylor J, Diop A, Blum J, Dolo O, Winikoff B (2011). Oral misoprostol as an alternative to surgical management for incomplete abortion in Ghana. Int J Gynecol Obstet..

[14] Schwekerela B, Kalumuna R, Kipingili R, Mashaka N, Westheimer E (2007). Misoprostol for treatment of incomplete abortion at the regional hospital level: results from Tanzania. BJOG..

[15] Fawole AO, Diop A, Adeyanju AO, Aremu OT, Winikoff B (2012). Misoprostol as first-line treatment for incomplete abortion at secondary-level health facility in Nigeria. Int J Gynecol Obstet..

[16] Shaw D (2007). Misoprostol for reproductive health: Dosage recommendations. Int J Gynecol Obstet..

[17] Tang OS, Ho PC (2006). Misoprostol: Pharmacokinetics and different regimens of misoprostol in early first-trimester medical abortion. Contraception..

[18] Mayi-Tsonga S, Meyé JF, Tagné A, Ndombi I, Diallo T (2007). Audit de la morbidité obstétricale grave « Near miss » au Gabon. Clin Mother Child Health..

[19] Lee DT, Cheung LP, Haines CJ, Chan KP, Chung TK (2001). A comparison of the psychologic impact and client satisfaction of surgical treatment with medical treatment of spontaneous abortion: a randomized controlled trial. Am J Obstet Gynecol..

[20] Shochet T, Diop A, Gaye A, Nayama M, Sall AB (2012). Sublingual misoprostol versus surgical care for treatment of incomplete abortion in five Sub-Saharan African countries. BMC Pregnancy Childbirth..

